# Unawareness of Apathy in Parkinson’s Disease: The Role of Executive Dysfunction on Symptom Recognition

**DOI:** 10.3390/brainsci13060964

**Published:** 2023-06-18

**Authors:** Gianpaolo Maggi, Carmine Vitale, Alessia Delle Curti, Marianna Amboni, Gabriella Santangelo

**Affiliations:** 1 Department of Psychology, University of Campania “Luigi Vanvitelli”, 81100 Caserta, Italy; alessia.dellecurti@studenti.unicampania.it (A.D.C.); gabriella.santangelo@unicampania.it (G.S.); 2 Institute of Diagnosis and Health, IDC-Hermitage Capodimonte, 80131 Naples, Italy; cavit69@hotmail.com (C.V.); marianna.amboni@gmail.com (M.A.); 3 Department of Motor Sciences and Wellness, University “Parthenope”, 80133 Naples, Italy; 4 Department of Medicine, Surgery and Dentistry, University of Salerno, 84084 Salerno, Italy

**Keywords:** anosognosia, awareness, apathy, Parkinson’s Disease, non-motor symptoms, quality of life

## Abstract

Altered self-awareness or anosognosia may impact patients’ everyday life by interfering with their safe and independent functioning. Symptom awareness has been linked to executive dysfunctions caused by damage to frontal regions. Apathy is a frequent neuropsychiatric manifestation of Parkinson’s disease (PD) and is considered a consequence of altered functioning of cortico-subcortical circuitries connecting the prefrontal cortex (PFC) with the basal ganglia. Thus, apathetic PD patients may be not be fully aware of their condition due to shared neuropathophysiological mechanisms. The present study aimed to explore the awareness of apathy in PD patients by comparing the self-reported evaluations with their caregivers’ ratings. Moreover, we explored the clinical predictors of possible discrepancies and their consequences on patients’ self-reported evaluation of quality of life (QoL). We found a fair agreement between patients’ self-reports and caregivers’ ratings on apathy scores, with patients reporting less severe apathetic symptoms, especially those related to executive and auto-activation processing, compared to their caregivers’ reports. Executive functioning was found to mediate the relationship between disease stage and awareness of the apathetic state. Awareness of executive apathy impacted patients’ self-reported QoL. Therefore, PD patients might be unaware of their apathetic symptoms, especially those with worse executive functioning, which plays a key role in metacognitive processes such as self-monitoring and error detection. Anosognosia for apathy in PD patients may affect their QoL perception and leads to misleading self-report evaluations that delay diagnosis and treatment.

## 1. Introduction

Accurate awareness of one’s own abilities is essential for optimal everyday functioning, as this capacity allows one to select the most appropriate activities according to one’s own possibilities and limitations [1]. Altered self-awareness or anosognosia, on the other hand, referring to difficulties in accurately estimating one’s own functional capacity [2], may lead to choosing activities not tailored to physical or mental capacities, resulting in dangerous behaviors toward oneself and others (e.g., driving, managing finances, cooking) that interfere with safe and independent functioning [3].

Anosognosia frequently occurs in neurodegenerative and neurological diseases that cause progressive impairments in several functional domains (i.e., motor, cognitive, affective, and social interpersonal abilities) [3]. Therefore, these patients may present an altered capacity to recognize their difficulties, such as denying impairments in memory or thinking domains, neuropsychiatric disturbances, and/or motor disabilities [2]. This profoundly affects patients’ care, leading to delayed diagnosis, resistance, reduced adherence to treatment, and the burden of caregivers who must constantly supervise patients to avoid them engaging in risky behaviors [1].

Awareness deficits have been consistently linked to frontal dysfunctions, possibly because of the functionally integrative role of the prefrontal cortex (PFC) [4]. More specifically, it has been demonstrated the crucial role of metacognition with self-monitoring and self-appraisal mechanisms is essential for anticipating, recognizing, and self-correcting errors during task performance [5]. Within neurodegenerative diseases, anosognosia and deficits of self-awareness have been frequently observed in Alzheimer’s dementia (AD), whose patients underestimate the extent of their memory loss, and in the behavioral variant of frontotemporal dementia (bvFTD), with patients showing loss of insight for their own behavioral and personality changes [6].

In non-demented PD patients, a reduced ability to recognize their own motor state has been reported regardless of functional impairment (e.g., ON state with and without dyskinesias, ON-OFF transition) [7,8]. However, PD patients may show altered awareness also for non-motor symptoms, including cognitive dysfunctions and neuropsychiatric manifestations, that seems to be modulated by motivation and emotional processing together with executive functioning [1,9].

Apathy, defined as a “simultaneous diminution in the cognitive and emotional concomitants of goal-directed behavior” [10], is a frequent neuropsychiatric manifestation of PD and is related to more severe cognitive dysfunctions [11,12], caregivers’ burden and worse patients’ quality of life (QoL) [13]. Multiple underlying mechanisms have been proposed to distinguish three different subtypes of apathy-disrupted processing: “emotional-affective”, “cognitive”, and “auto-activation” [14]. This different processing of apathy refers to specific cortico-subcortical circuitries that connect PFC with basal ganglia [14]. It has been hypothesized a relationship between anosognosia in neurodegenerative disease and apathy since altered emotional reactivity related to task performance may impact motivation, that in turn affects the degree of active monitoring during task performance [1]; thus, apathy in PD patients may also affect the awareness of their own condition due to shared neuropsychological (reduced self-monitoring) and neuropathophysiological (altered functioning of PFC) mechanisms [1,9]. Moreover, apathy in PD has been linked to worse cognition, especially executive functioning [11,12], which is crucial for the comprehension of one’s own (self-reflectivity) and others’ mental states (other-reflectivity) [15,16,17]. Nevertheless, studies on awareness of neuropsychiatric symptoms in PD have yielded mixed results. A study by Mathias and colleagues [18] found a satisfactory agreement between patients’ and informants’ ratings on neuropsychiatric scales except for depressive symptoms that informants rated as more severe compared to patients’ reports; as for apathy, a study by Valentino and collaborators [19] showed that PD patients overestimated their apathy symptoms with respect to their caregivers’ ratings. Finally, Schiehser and colleagues [20] demonstrated a substantial agreement between the two ratings.

Considering that the possible under/over-estimation of apathy by patients may affect their identification and management and influence both QoL and overall prognosis, the present study aimed to explore the degree of awareness for each subtype of apathy in non-demented PD patients comparing self-reported evaluations with their caregivers’ ratings. Furthermore, we examined the possible clinical predictors of discrepancy in apathy scores and whether and how reduced awareness of apathy impacts self-reported QoL.

## 2. Materials and Methods

### 2.1. Participants

Consecutive PD outpatients referred to the Movement Disorders Unit of IDC-Hermitage Capodimonte (Naples, Italy) were screened. PD patients were included in the study whether they met the following inclusion criteria: i. a diagnosis of idiopathic PD according to the clinical diagnostic criteria; ii. absence of cognitive decline by means of the Italian version of the Montreal Cognitive Assessment (MoCA > 15.5 [21]); iii. absence of neurodegenerative or cerebrovascular disorders other than PD; iv. absence of major depression according to DSM-5 diagnostic criteria; v. the presence of a cognitively unimpaired caregiver able to provide an informant report of the patient’s symptoms of apathy.

Demographic (i.e., age, sex, educational level) and clinical aspects (i.e., disease duration, severity of motor symptoms assessed by both the Hoehn and Yahr staging system (H&Y) and part III of the Unified Parkinson’s Disease Rating Scale (UPDRS), Levodopa Equivalent Daily Dose (LEDD), and depressive symptomatology evaluated using the Beck Depression Inventory-II (BDI-II) [22] were recorded. Patients underwent the clinical visit during the ON phase.

All participants gave their written informed consent to participate in the study, which was approved by the Local Ethics Committee and was performed in accordance with the ethical standards laid down in the 1964 Declaration of Helsinki and its later amendments.

### 2.2. Neuropsychological Evaluation

#### 2.2.1. Apathy

Apathy was evaluated using the Italian version of the Dimensional Apathy Scale (DAS) [23,24], a questionnaire validated in PD [23] for the assessment of apathetic symptomatology, minimizing the impact of motor symptoms in both SE and CR versions. This questionnaire consists of 24 items rated on a 4-point Likert scale and three subscales: i. executive subscale assessing apathetic symptoms related to impaired executive processes necessary to achieve goals, including planning, organization, and attention monitoring; ii. emotional subscale assessing apathy associated with diminished integration, processing, and expression of emotional behaviors resulting in a lack of affectivity; and iii. behavioral/cognitive initiation subscale assessing apathy associated with loss of cognitive and behavioral initiation. The total score ranges from 0 to 72, with higher scores indicating more severe apathy. A cut-off score of 29 was employed to determine the occurrence of apathy [25].

Patients and their caregivers completed DAS. All caregivers were patients’ relatives who routinely cared for them and lived in the same household. None of participants had cognitive impairments or significant psychiatric disorders, and they were previously informed of the aims of the study.

#### 2.2.2. Cognitive Evaluation

All participants underwent a neuropsychological assessment covering different aspects of executive control [26,27,28], such as cognitive flexibility and set-shifting (i.e., phonological verbal fluency task and part B-A of the Trail Making Test), inhibitory control (i.e., Color-word Interference task of the Stroop test), and processing speed/attention/working memory tests (i.e., the Color reading task of the Stroop test and part A of the Trail Making Test). Adjusted scores for each executive measure were converted into z-scores using the available normative data. Then, a composite score, named executive control, was calculated by integrating z-scores on each executive test.

### 2.3. Statistical Analysis

We calculated descriptive statistics about the demographic, clinical, and neuropsychological variables of the sample.

To evaluate the agreement between patients’ self-reports and their caregivers’ ratings on the occurrence of apathy (apathetic vs. non-apathetic) [24], Cohen’s kappa (κ) was calculated. The level of agreement was interpreted as follows: κ < 0.00 poor; 0.00 ≤ κ ≤ 0.20 slight; 0.21 ≤ κ ≤ 0.40 fair; 0.41 ≤ κ ≤ 0.60 moderate; 0.61 ≤ κ ≤ 0.80 substantial; 0.81 ≤ κ ≤ 1.00 almost perfect [29]. Moreover, repeated-measures analysis of variance (ANOVA) was performed to compare PD patients’ self-reports and caregivers’ ratings on the DAS scores.

Both patients’ self-report and caregivers’ rating scores were converted into Z scores based on Italian normative data [24], with higher values indicating worse apathetic symptoms. Discrepancy scores for the DAS total score and its subscales were calculated by subtracting self-reported z-scores from the respective caregiver z-scores. Thus, positive discrepancy scores indicate patients’ underestimation and negative discrepancy scores indicate patients’ overestimation of apathetic symptoms.

Moreover, to explore the clinical predictors of a discrepancy, we carried out multiple regression analyses entering the discrepancy scores for the DAS total score and its subscales as dependent variables and age, UPDRS-III, H&Y, LEDD, and executive control score as predictors. In the same way, to investigate whether and how discrepancy scores affected the self-reported quality of life scores, multiple regression analyses entered clinical variables and discrepancy scores as independent variables and QoL (PDQ-8) as dependent variables.

Subsequently, designed mediation models were tested to examine the exact nature of these relationships. The bootstrapping procedure with 5000 samples and replacement from the full sample was used to construct bias-corrected 95% confidence intervals (hereafter 95% CI; LL = lower level of the confidence interval, UL = upper level of confidence interval). Mediation models were carried out using SPSS Macro PROCESS [30]. The significance level was set at α = 0.05, and all statistical analyses were performed using SPSS Statistic 26.0.

## 3. Results

We included 67 PD patients. Descriptive statistics about demographic, clinical, and neuropsychological are reported in Table 1.

### 3.1. Agreement between Patients’ Self-Report Apathy Scores and Caregivers’ Ratings

**Figure 1:**
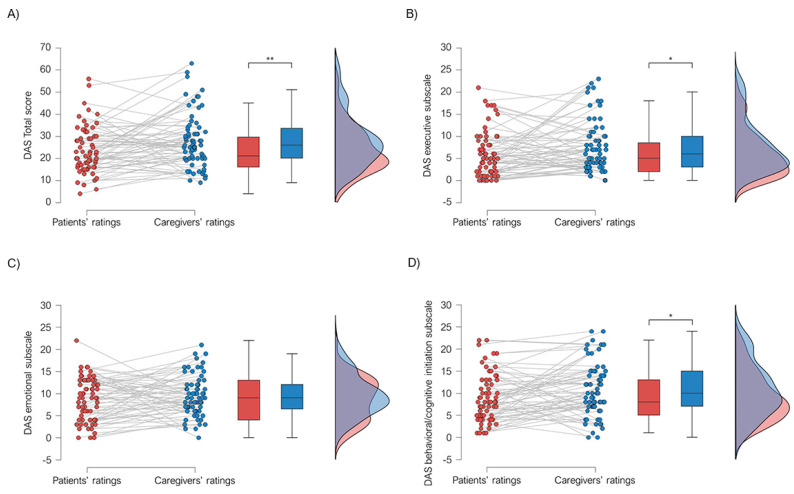
Raincloud plot representing discrepancies between patients’ and caregivers’ ratings on (**A**) DAS Total score, (**B**) DAS executive subscale, (**C**) DAS emotional subscale, and (**D**) DAS behavioral/cognitive initiation subscale. Clouds represent distribution, raindrops represent individual participants, and bars represent 95% confidence intervals. (* *p* < 0.05; ** *p* < 0.01).

We found a fair agreement between patients’ and their caregivers’ ratings on the occurrence of apathy (κ =  0.251, *p* = 0.037). A significant difference between patients’ and caregivers’ ratings emerged on the DAS total score (F(1, 66) = 8.250; *p* = 0.005; η^2^ = 0.111), with caregivers reporting higher scores (mean = 28.07; SE = 1.50) than patients (mean = 23.51; SE = 1.29; Figure 1A). We found a significant difference between patients’ and caregivers’ ratings on the DAS executive subscale (F(1, 66) = 5.686; *p* = 0.020; η^2^ = 0.079), with caregivers reporting higher scores (mean = 7.75; SE = 0.71) than patients (mean = 5.85; SE = 0.64; Figure 1B); moreover, caregivers reported higher ratings on the DAS behavioral/cognitive initiation subscale than patients (F(1, 66) = 5.828; *p* = 0.019; η^2^ = 0.081) (caregivers ratings: mean = 10.84; SE = 0.74; patients ratings: mean = 9.01; SE = 0.66; Figure 1D). Conversely, no significant difference between patients’ and caregivers’ ratings on the DAS emotional subscale (F(1, 66) = 1.636; *p* = 0.205; η^2^ = 0.024; Figure 1C).

### 3.2. Clinical Predictors of Discrepancy between Patients’ Self-Report Apathy Scores and Caregivers’ Ratings

Multiple regression analysis revealed that higher discrepancies in DAS total scores (patients’ underestimation of apathy) were related to less severe H&Y stage (B = −0.844, t = −2.702, *p* = 0.009) and lower executive control scores (B = −0.077, t = −2.638, *p* = 0.011). Moreover, lower H&Y scores were associated with a higher discrepancy on the DAS executive subscale (B = −0.802, t = −2.153, *p* = 0.035), while higher UPDRS-III scores were associated with a higher discrepancy on the DAS behavioral/cognitive initiation subscale (B *=* 0.045, t *=* 2.339, *p* = 0.023). No significant association was found between the discrepancy on the DAS emotional subscale and the clinical variables.

Subsequently, a mediation model was designed to explore the nature of the relationship between H&Y stage, executive control, and the discrepancy in the DAS total score. We found that a less severe H&Y stage was related to higher executive control (B = −3.624; *p* < 0.001), and consequently, lower H&Y (B = −0.700, t = −3.244, *p* = 0.002) and poorer executive control (B = −0.068, t = −2.375, *p* = 0.021) were related to a higher discrepancy on the DAS total scores (patients’ underestimation of apathetic symptoms). Examining the indirect effects, using the bootstrap-generated bias-corrected CI approach, a significant indirect effect of the H&Y stage on discrepancy through executive control was found (Estimate effect: 0.246; 95% CI: 0.065–0.506). Thus, better patients’ executive control mediated the effect of H&Y stage, leading to less discrepancy in apathy ratings, while worse executive control mediated the effect of H&Y stage, producing a more pronounced discrepancy in apathy ratings.

### 3.3. Effect of Discrepancy on Self-Report Quality of Life

Multiple regression analysis indicated that more severe H&Y stage (B = 5.141, t = 2.766, *p* = 0.008) and less discrepancy on the DAS executive subscale (patients’ overestimation of apathy) (B = −1.415, t = −2.212, *p* = 0.031) were associated with poorer QoL.

**Figure 2:**
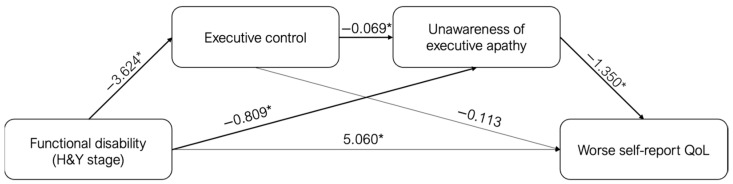
Scheme of the mediation effects (represented by bold lines) of executive control and Unawareness of executive apathy in the relationship between H&Y stage and worse self-report QoL. (* *p* < 0.05).

Subsequently, considering the previous mediation model, the final model was tested to examine the impact of discrepancy on apathy on self-reported QoL scores. We found that poorer QoL scores were linked to a more severe H&Y stage (B = 5.060, t = 3.850, *p* < 0.001) and lower discrepancy on the DAS executive subscale (patients’ overestimation of apathy) (B = −1.350, t = −2.239, *p* = 0.029), but not to executive control (B = −0.113, t = −0.677, *p* = 0.510). Analyses of the indirect effects revealed a significant indirect effect of H&Y stage on self-reported QoL through discrepancy on executive subscale (Estimate effect: 1.092; 95% CI: 0.104–2.487) and of H&Y stage on QoL self-report scores through a mediation pathway involving executive control and discrepancy on the DAS executive subscale (H&Y → Executive control → Discrepancy on Executive subscale → PDQ-8) (Estimate effect: −0.338; 95% CI: −0.989–−0.007). Therefore, the less severe H&Y stage associated with more preserved executive control and less discrepancy in executive apathy ratings generated poorer ratings of QoL. In contrast, the more severe H&Y stage related to worse executive control and more discrepancy in executive apathy ratings resulted in better QoL ratings (Figure 2).

## 4. Discussion

In the present study, we evaluated patients’ awareness of their apathetic symptoms by comparing self-reported scores with caregivers’ ratings. Moreover, we unraveled the clinical predictors of discrepancy scores and how altered awareness affects self-reported QoL ratings.

We found fair agreement between patients’ self-reports and caregivers’ ratings of apathy. Particularly, patients reported less severe apathetic symptoms, especially those related to executive and auto-activation processing. Apathy due to disruption of “cognitive” processing relies on a decrease in the cognitive resources needed to elaborate and execute the plan of goal-directed behavior (also defined as “cognitive inertia”) [14]. Cognitive inertia has been associated with dysfunction of the associative cortico-basal network connecting the dorsal portion of the caudate nucleus with the dorsolateral prefrontal cortex (DLPFC), which also accounts for cognitive dysfunctions, especially in the elaboration, execution, and control of goal-directed behaviors [11,14,31]. This dimension is measured by the executive subscale of DAS by items evaluating patients’ planning abilities, attention monitoring, and concentration [23].

In contrast, apathy related to an “auto-activation” deficit results from a failure to reach the threshold of initiation/activation of thoughts or actions on an internal basis and can be reversed by external stimulation (“hetero-activation”). Alterations in the dorsal-medial regions of the PFC, including the supplementary motor area (SMA) and the dorsal part of the anterior cingulate cortex (ACC), have been related to “auto-activation” deficits [14]. This dimension is measured by items of DAS referring to questions contrasting self- and externally driven behaviors in activities of daily living, such as “Do you need a push to get started on things?” [23].

Taking this into account, the lack of awareness of PD patients in these dimensions of apathy might be explained by common neuropathophysiological mechanisms involving both the DLPFC and ACC, which exert a pivotal role in the online monitoring of performance and error detection [32,33,34,35]. In summary, it is possible to hypothesize that PD patients tend to underestimate their apathetic state due to a failure in error monitoring. Considering that the degree of monitoring is affected by motivational factors since emotional reactivity marks the salience of an event [36], errors and their consequences are often ignored by apathetic PD patients because apathy interferes with their ability to feel affective signals downplaying the emotional impact of errors [37].

Nevertheless, a dissociation emerged between patients’ unawareness of apathy, expressed by the executive and initiation dimensions, and their preserved emotional reactivity resulting from the DAS emotional subscale. These findings are in line with previous studies conducted in the AD population, showing that AD patients are not prone to awareness deficits of emotional aspects of functioning, manifesting appropriate emotional reactivity to the experience of failure in cognitive tasks despite limited awareness of the condition or performance [37,38].

The evaluation of possible clinical predictors of discrepancy scores revealed that worse executive control and less severe functional disability (evaluated by H&Y) were associated with greater discrepancy scores. Interestingly, an inverse relationship between unawareness of motor symptoms and disease severity has been observed in PD [39,40], leading to the hypothesis that patients lose the ability to recognize their own motor manifestations until these interfere with a specific task, reaching conscious awareness [39]. Similarly, patients’ underestimation of their apathetic symptoms might be explained by impaired self-monitoring, linked to aberrant functioning of the DLPFC and ACC [32,33,35], and then worsened by low functional interference in everyday life.

Within this context, executive functioning emerged as a mediator of this relationship, with worse executive control associated with unawareness of the apathetic state. Executive models of self-awareness focus on the role of higher-order executive processes that regulate self-monitoring mechanisms and relate to several metacognitive abilities, such as recognition of deficits, understanding their functional implications, and setting realistic goals accordingly [41,42]. Altered symptom awareness in PD has been attributed to an aberrant functioning of the fronto-striatal circuits with the involvement of executive dysfunctions and impaired working memory [9]. Indeed, the spread of PD neurodegenerative processes towards the cortico-striatal and meso-cortical dopaminergic circuits involves the medial PFC [43,44], which plays a pivotal role in metacognition, especially in the judgment of performance and error detection [33,34,35]. Recognition of discrepancies between actual and expected performance promotes the adjustment of performance and/or the selection of alternative strategies, which in turn leads to further restructuring of one’s own knowledge and beliefs.

Moreover, exploring whether and to what extent discrepancy scores on the DAS subscales can predict the level of QoL reported by patients, we found that poorer patients’ QoL ratings were affected by more severe PD stage/functional disability (H&Y) and more awareness of executive apathy. More specifically, awareness of executive apathy mediated the relationship between functional disability and subjective QoL reports, with greater awareness associated with worse QoL. To this, our findings suggest that executive control and awareness of executive apathy mediate the relationship between disease stage/functional disability and subjective QoL reports (H&Y -> executive control -> awareness of executive apathy -> subjective QoL reports), further highlighting the inaccuracy of apathetic symptoms and QoL when subjectively reported by patients. Paradoxically, patients in a more severe PD stage and with poorer executive control were unaware of their apathetic symptoms, scoring better QoL; on the contrary, patients in a less severe PD stage and with preserved executive functioning were more aware of their apathetic symptoms and reported worse QoL. Overall, our findings confirm the key role of executive functioning, especially self-monitoring, in symptom awareness in PD and suggest the need to investigate neuropsychiatric aspects such as apathy not only through self-report measures but also using an informant version of tools, since patients might not be aware of their condition.

However, some limitations should be addressed. First, subjective features such as personality traits and psychological burden may impact patients’ reports, resulting in under/overestimation of symptoms. Moreover, it should be considered that the knowledge each caregiver has of patients’ symptomatology may vary across participants; however, in our study, we identified the person most involved in patients’ everyday life and care. To this, the development of objective measures such as performance-based tools [45] and the analysis of behavioral data [46] may help clinicians overcome these limitations and obtain more reliable measurements.

## 5. Conclusions

In conclusion, the present study aimed to investigate PD patients’ apathetic symptoms also through informant-report measures, especially in those with poorer executive functioning, as they might be unaware of their condition. Unawareness of apathy in PD patients provokes frustration in their caregivers and family members who must assist resistant patients, but also has profound implications for themselves as it can delay the diagnosis and implementation of targeted treatment strategies and ultimately affect the overall prognosis.

## Figures and Tables

**Table 1:** Descriptive statistics on demographic, clinical, and neuropsychological variables.

	Mean ± SD
Age (ys)	66.06 ± 8.03
Education (ys)	11.54 ± 4.48
Gender (n)	M = 44; F = 23
Disease Duration (ys)	9.57 ± 5.93
UPDRS-III	14.84 ± 8.60
Hoehn and Yahr	2.35 ± 0.63
LEDD	732.21 ± 413.17
MoCA	19.82 ± 4.47
BDI-II	7.28 ± 6.79
TMT: A	73.88 ± 50.94
TMT: B-A	179.67 ± 124.90
Phonological fluency	28.64 ± 10.82
Stroop Test-Color	33.87 ± 12.79
Stroop Test-Interference	12.85 ± 8.09

SD, Standard deviation; ys, years; n, number; UPDRS, Unified Parkinson’s Disease Rating Scale; LEDD, Levodopa Equivalent Daily Dose; MoCA, Montreal Cognitive Assessment; BDI-II, Beck Depression Inventory–II; TMT, Trail Making Test.

## Data Availability

Data will be available under request.
